# Efficacy and Safety of Veverimer in the Treatment of Metabolic Acidosis Caused by Chronic Kidney Disease: A Meta-analysis

**DOI:** 10.3389/fphar.2021.643128

**Published:** 2021-04-29

**Authors:** Wenlin Liu, Lili Li, Xuemei Zhang, Haonan Dong, Miaomiao Lu

**Affiliations:** Department of Nephrology, The First Affiliated Hospital of Jinzhou Medical University, Jinzhou, China

**Keywords:** metabolic acidosis, chronic kidney disease, serum bicarbonate, veverimer, meta-analysis

## Abstract

Metabolic acidosis is a common complication of chronic kidney disease (CKD). Veverimer is an orally administrated, free amine polymer with high capacity and binding selectivity to hydrochloric acid from the gastrointestinal tract. This study pooled the current evidence of the efficacy and safety of veverimer for the treatment of metabolic acidosis associated with CKD. We conducted a systematic literature search on PubMed, Embase, and Cochrane Central for relevant randomized controlled trials (RCTs) in June 2020. In this study, three RCTs with 548 patients were included in our analysis. The analysis revealed that veverimer was associated with increased bicarbonate level of patients (weight mean difference [WMD] 3.08, 95% confidence interval [CI] [2.40, 3.77], *p* < 0.001) and improved physical function compared with placebo measured by Kidney Disease and Quality of Life Short Form 36, question 3 (physical functioning domain) (KDQoL-PFD) score (WMD 5.25, 95% CI [1.58, 8.92], *p* = 0.005). For safety outcomes, both groups exhibited similar risks for developing headache, diarrhea, flatulence, and hyperkalemia. In conclusion, current clinical evidence indicates that veverimer is efficacious and safe against metabolic acidosis related to CKD compared with placebo. Further research comparing long-term veverimer use with traditional alkali therapy is needed.

## Introduction

Chronic kidney disease (CKD) is a long-term structural or functional disorder of the kidneys, manifested by elevated serum levels of creatinine, cystatin C, or blood urea nitrogen (Drawz and Rahman, 2015). The estimated prevalence rate for all stages of CKD was 13% (Hill et al., 2016). Most patients with CKD are at risk for accelerated cardiovascular disease and death, with significantly impaired life expectancy and quality of life.

Metabolic acidosis is a common and persistent complication of CKD, which contributes to the continued progression of CKD. It is associated with a decrease in total renal ammonium excretion, titratable acid excretion, and bicarbonate reabsorption as a result of a decline in glomerular filtration rate (GFR) (Kraut and Madias, 2016). Previous studies have shown that chronic disturbances in serum acid excretion with increased serum bicarbonate are related to increased risk for renal disease progression, heart failure, and all-cause mortality (Shah et al., 2009; Menon et al., 2010; Raphael et al., 2011; Dobre et al., 2013).

Traditional treatments of metabolic acidosis mainly involve oral alkali supplementation. However, previous evidence demonstrated that oral alkali supplementation might result in edema, atherosclerosis progression (Lomashvili et al., 2006). In addition, the management of dietary interventions was proved difficult to fulfill, resulting in suboptimal treatment outcomes.

Veverimer (TRC101) is a novel oral and nonabsorbable hydrochloric acid adhesive. It is not an ion exchanger and does not introduce sodium ions. The structure and nature of veverimer dictate that it is protonated upon ingestion and selectively binds to anions, resulting in a reduction and removal of hydrochloric acid from the gastrointestinal tract (Wesson et al., 2019b; Adrogué and Madias, 2020). Data obtained from clinical trials (Bushinsky et al., 2018; Wesson et al., 2019a; Wesson et al., 2019b) have revealed that veverimer was effective against acidosis related to CKD, thereby improving patients’ bicarbonate level. The endogenous increase in serum bicarbonate without sodium retention is a unique feature of veverimer. However, some researchers have found that the mechanism of veverimer parallels the persistent vomiting to remove gastric acid. The difference is that add bicarbonate level of persistent vomiting is accompanied by the loss of endogenous fluid and ions; whereas the increased endogenous bicarbonate with veverimer does not lose fluid, sodium and potassium, etc. (Adrogué and Madias, 2020). No systematic approach has been conducted to evaluate the safety and efficacy of veverimer for the treatment of metabolic acidosis associated with CKD. Therefore, we conducted a meta-analysis to analyze the efficacy and safety veverimer.

## Methods

### Search Strategy

We conducted a systematic search for relevant studies evaluating the use of veverimer for the treatment of acidosis in patients with CKD. This meta-analysis protocol was performed in strict accordance with the preferred reporting items for systematic reviews and meta-analysis (PRISMA) statement (Moher et al., 2009). MEDLINE (via PubMed), Embase, and the Cochrane Central Register of Controlled Trials were searched from 2000 to June 2020. The search was conducted using Medical Subject Headings (MeSH) terms and the keywords “veverimer,” “CKD,” “chronic renal disease,” “chronic kidney disease,” “kidney failure, chronic,” and “metabolic acidosis.” In addition, the relevant trials were accessed using the ClinicalTrials.gov platform. Related references were screened to avoid omission. There were no language restrictions.

### Eligibility Criteria

Inclusion criteria: 1) Randomized controlled trials (RCTs); 2) Studies evaluating veverimer use in adult subjects (≥18 years) with acidosis and CKD. Metabolic acidosis refers acid-base disturbance, which caused by increased H^+^ or loss of HCO3^−^ in extracellular fluid {[HCO3^−^] <22 mmol/L} (Raphael, 2019; Gone and Chen, 2020). CKD is defined as a progressive disease of the kidneys with structural and functional disorders or GFR lower than 60 ml/(min·1.73 m^2^) for at least 3 months (Peng et al., 2019), patients of chronic renal insufficiency often leads to high anion gap (AG) acidoses; 3) Patients randomly assigned to experimental and control group. Exclusion criteria: reviews, studies with insufficient data, and patients with normal serum bicarbonate were excluded from the analysis. All eligible studies had a comparison of veverimer with the placebo group.

### Quality Assessment

Two authors independently used in accordance with the Cochran Collaboration risk of bias tool for evaluating the quality of RCTs as described in a previous study (Higgins et al., 2011), following selection bias, performance bias, detection bias, attrition bias, reporting bias were assessed. In brief, these items were rated as “low risk”, “high risk”, or “unclear” evaluating individual study. Discrepancies regarding the quality assessment were solved by discussion with another experienced investigator.

### Extraction of Data and Outcome

Two review authors (WL and LL) extracted data from these studies, including the following information: 1) first author, 2) number of subjects, 3) country, 4) recruitment period, 5) study design, 6) mean age of patients, 7) sex, 8) primary outcomes, 9) follow-up time (10) inclusion criteria, and 11) national clinical trial number. In addition, we summarized the baseline characteristics of the study population. Any disagreements regarding the inclusion or exclusion of the articles were resolved by consensus or adjudicated by the third reviewer (ML).

For the efficacy outcome, changes in bicarbonate concentration over time were the primary endpoint. The secondary efficacy endpoints include Kidney Disease and Quality of Life Short Form (SF) 36, question 3 (physical functioning domain) (KDQoL-PFD) score, and repeated chair stand (s). In addition, we evaluated the adverse events of veverimer in patients, which include diarrhea, headache, flatulence, hyperkalemia, the decreased level of GFR and influenza.

### Statistical Analysis

Review Manager (version 5.3) was used for all data analyses in this study. Enumeration data were used to analyze the risk ratio and measurement data to obtain the weighted mean differences (WMDs) to compare the endpoints of the two groups. For each effect size, we provide values and 95% confidence intervals (CIs). *p* < 0.05 was considered statistically significant. Besides, I^2^ index was performed to evaluate heterogeneity. I^2^ > 50% and *p* < 0.1 were considered to indicate significant heterogeneity, and the random-effect model was selected, while I^2^ < 50% and *p* > 0.1 represent good homogeneity, and the fixed-effect model was chosen for analysis. Therefore, the meta-analysis was performed using the fixed-effect model with the DerSimonian and Laird method.

## Results

### Literature Search Results

**FIGURE 1:**
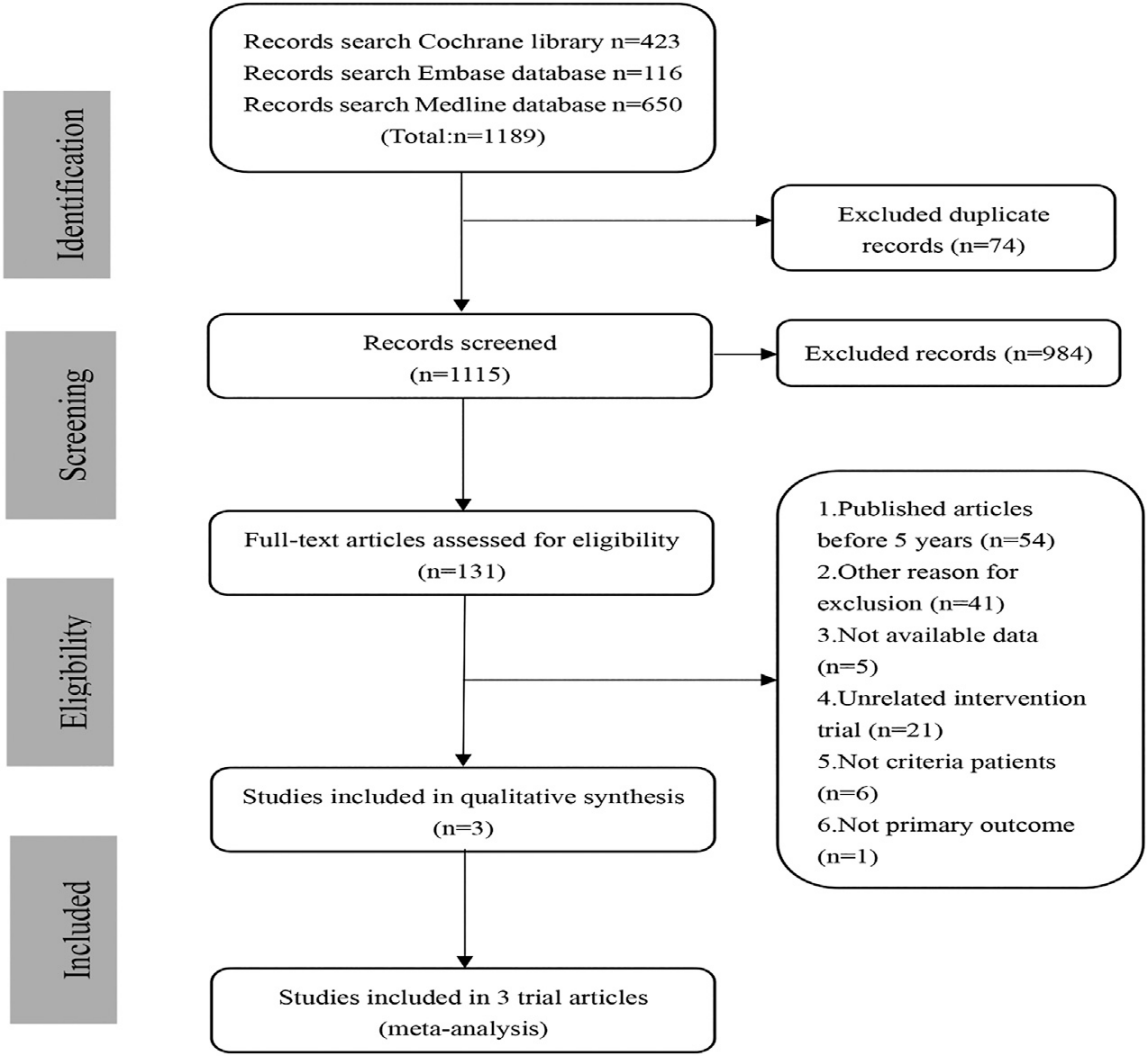
Flowchart for data extraction.

The search and screen process was illustrated in Figure 1. Literature search to 1,189 studies. After excluding 74 duplicate studies and 984 non-RCT studies, 131 articles remained. Of these 131 studies, three double-blinded RCTs (involving 135 patients randomly assigned to the phase 1/2 study, 217 patients randomly rationed to the 12 week-clinical trail, and 114 patients and 82 patients to veverimer *vs.* placebo in extension phase, respectively) (Bushinsky et al., 2018; Wesson et al., 2019a; Wesson et al., 2019b) were included in the quantitative analysis.

### Basic Characteristics and Quality Assessment of Studies

**FIGURE 2:**
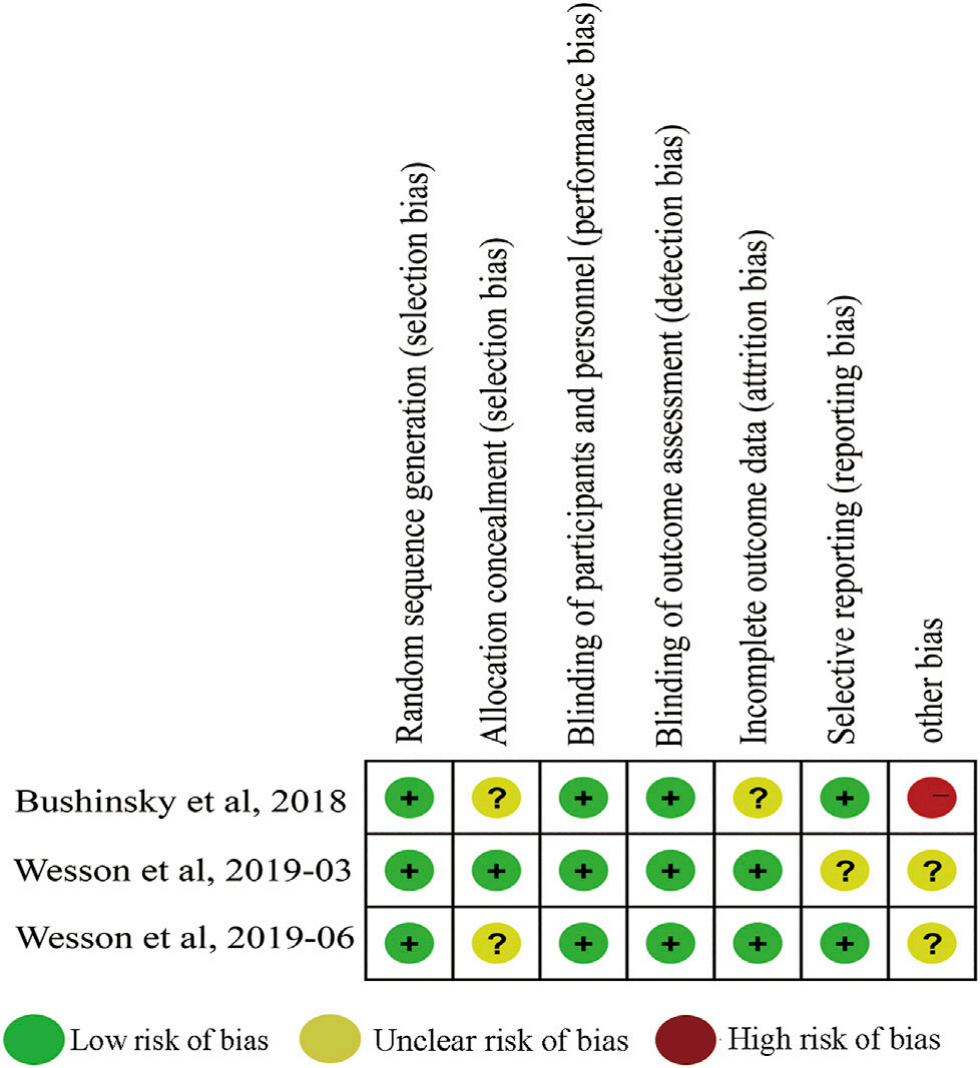
Bias risk assessment for inclusion in the study.

The baseline characteristics of the included studies are presented in Table 1. Notably, the 548 participants in the three trials were patients with acidosis, with 342 in the treatment group and 206 in the control group. The population of criteria for initiation was 12–20 mmol/L for [HCO3^−^] at hospitals and specialty clinics in Bulgaria, Georgia and other countries. Participants were treated with veverimer-placebo for 2, 12 and 52 weeks. Available data about changing from baseline in bicarbonate, physical function and safety assessment were obtained. The baseline characteristics of the studies are presented in Table 2. All of the studies were at low risk of bias (Figure 2).

**TABLE 1:** Characteristics of included randomized clinical trials.

References	Patients, *n*	Country	Recruitment period	Design and setting	Mean age of patients, years (SD)	Sex (male, female)	Primary outcome	Duration of follow-up (week)	Criteria for veverimer-placebo initiation	No of subjects
Wesson et al, 2019‐06	196	Bulgaria, Georgia, Hungary, Serbia, Slovenia, Ukraine, USA	2017–18	Multicentre, CKD and metabolic acidosis patients	Veverimer 62.9 (12.1); Placebo 61.7 (11.9)	61%; 39%	Long-term safety	40	eGFR 20–40 ml/min per 1.73 m^2^, serum bicarbonate 12–20 mmol/L	NCT03390842
Wesson et al, 2019‐03	217	Bulgaria, Georgia, Hungary, Serbia, Slovenia, Ukraine, USA	2017–18	Multicentre, CKD and metabolic acidosis patients	Veverimer 62.9 (12.6); Placebo 63.2 (12.1)	62.5%; 37.5%	Change from baseline in blood bicarbonate	12	eGFR 20–40 ml/min per 1.73 m^2^, serum bicarbonate 12–20 mmol/L	NCT03317444
Bushinsky et al, 2018	135	Bulgaria, Georgia	2016–20	Stage 3 or 4 CKD and metabolic acidosis patients	Veverimer 60 (13); Placebo 61 (12)	64%; 36%	Change from baseline to the end of treatment (Day 15) in serum bicarbonate within each individual TRC101 dose group	2	Patients had a mean baseline eGFR of 35 ml/min per 1.73 m^2^, a mean baseline serum bicarbonate of 17.7 mmol/L	NCT02809183

**TABLE 2:** Baseline characteristics of patients in the meta-analysis trials.

	Veverimer (*n* = 342)	Placebo (*n* = 206)
Age, years	61.9 (12.6)	61.9 (12.0)
Sex		
Male	209/342 (61%)	130/206 (63%)
Female	133/342 (39%)	76/206 (37%)
Hypertension	326/342 (95%)	199/206 (97%)
Left ventricular hypertrophy	145/342 (42%)	81/206 (39%)
Serum bicarbonate (mmol/L)	17.4 (1.3)	17.3 (1.5)
>18 mmol/L	80/238 (34%)	55/175 (31%)
≤18 mmol/L	158/238 (66%)	120/175 (69%)
Diabetes mellitus	220/342 (64%)	142/206 (69%)
Estimated GFR (ml/min per 1.73 m^2^)	31.2 (8.6)	29.9 (8.3)
KDQOL SF-36 physical function domain total score^a^	53.0 (23.0)	54.9 (26.7)
Repeated chair stand (s)^b^	19.5 (14.3)	18.3 (13.0)

Data are mean (SD) or *n* (%).

a Veverimer *n* = 237; placebo *n* = 175.

b Veverimer *n* = 225; placebo *n* = 160.

### The Efficacy of Veverimer Compared With Placebo

**FIGURE 3:**
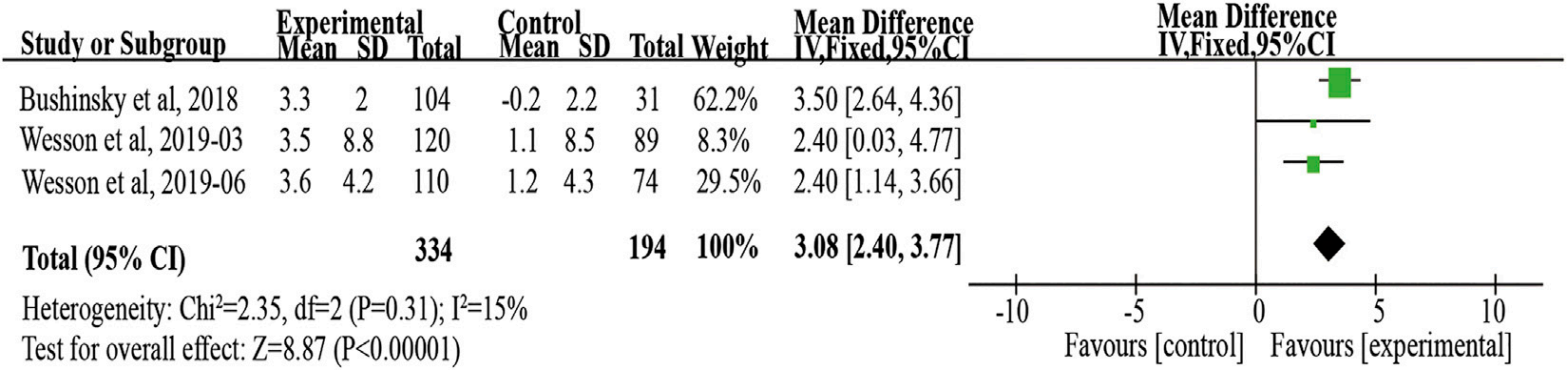
Forest plot for change in serum bicarbonate. IV, independent variable method; 95% CI, 95% confidence interval.

**FIGURE 4:**
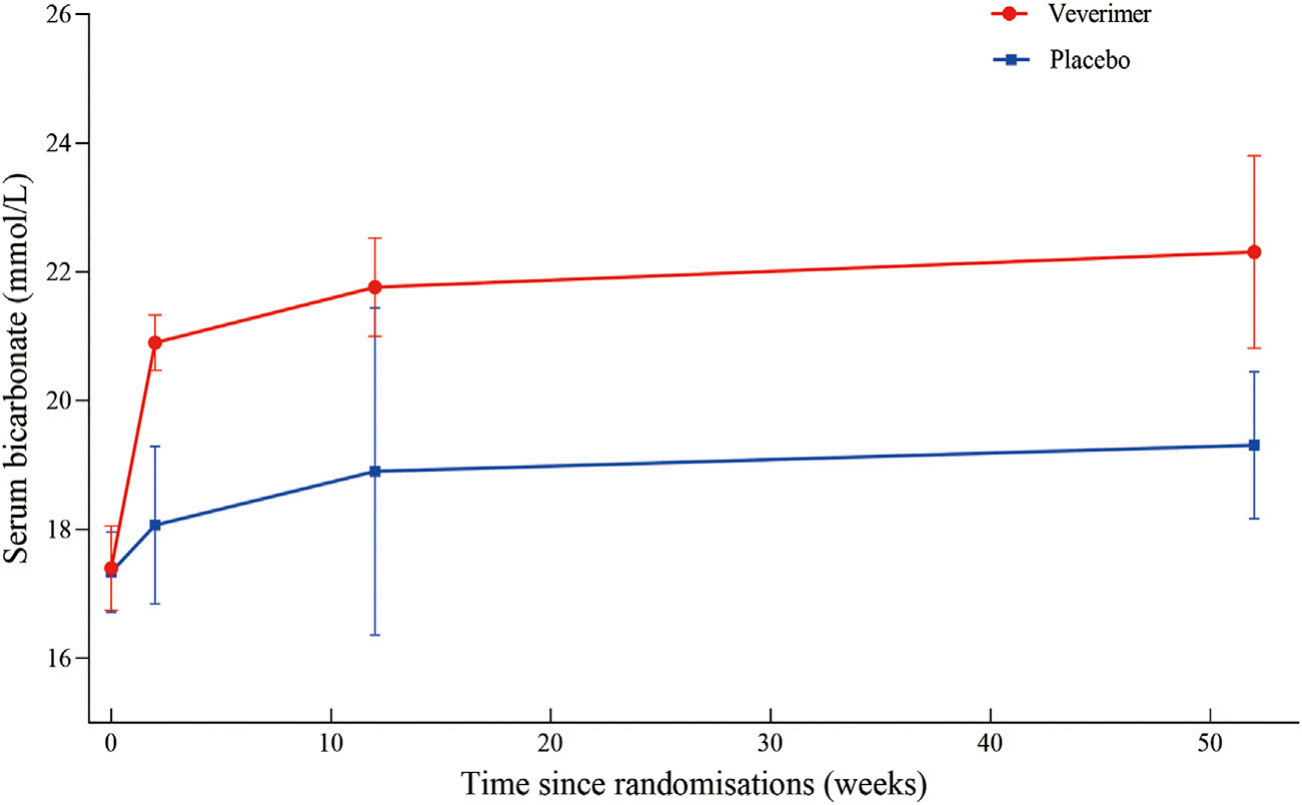
Changes in bicarbonate levels in the two groups during the 52 weeks.

**FIGURE 5:**
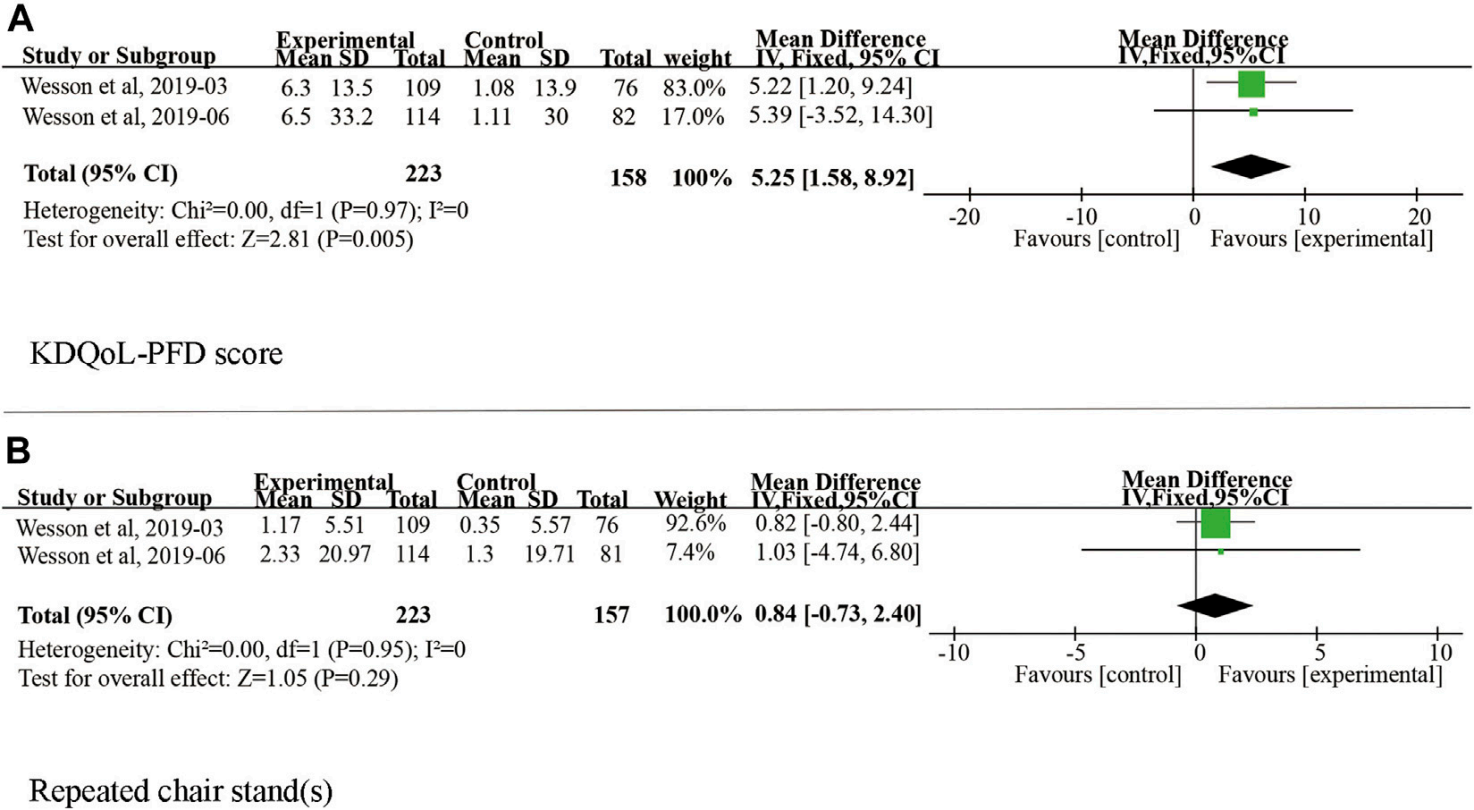
Forest plot for change in physical function. **(A)** Difference in KDQoL-PFD score among studies included in the meta-analysis. **(B)** Difference in repeated chair stand (s) among studies included in the meta-analysis. IV, independent variable method; 95% CI, 95% confidence interval.

Twenty patients were excluded from the primary outcome because of missing basic bicarbonate data. Among the remaining 528 subjects, the use of veverimer was associated with significantly increased serum bicarbonate level compared with the control group (WMD 3.08, 95% CI [2.40, 3.77], *p* < 0.001), and the heterogeneity was low (I^2^ = 15%, *p* > 0.05) (Figure 3). Regarding the subgroup indicators affecting the primary outcome, Wesson et al. (2019b) found that there was a significant improvement in [HCO3^−^] on average in the age group, sex, baseline bicarbonate, and screening eGFR groups of patients who took veverimer treatment, however this performance was not present in receiving acid reducing drug or alkali therapy patients. Figure 4 illustrates the change in bicarbonate concentration between the groups during the 52 weeks. These data also indicated that veverimer use was associated with persistent correction of bicarbonate level compared with the placebo group. In addition to the primary endpoint, pooled analysis of two studies (Wesson et al., 2019a; Wesson et al., 2019b) involving 381 participants revealed that veverimer significantly improved KDQoL-PFD score compared with placebo (WMD 5.25, 95% CI [1.58, 8.92], *p* < 0.01) with low heterogeneity (I^2^ = 0, Z = 2.81, *p* = 0.97) (Figure 5A). For repeated chair stand test time, after pooling data from two trials (Wesson et al., 2019a; Wesson et al., 2019b) involving 380 participants, veverimer was not associated with improved repeated chair stand(s) (WMD 0.84, 95% CI [−0.73, 2.40], *p* = 0.29) with low heterogeneity (I^2^ = 0, *p* = 0.95) (Figure 5B).

### The Safety Outcomes of Veverimer Compared With Placebo

**FIGURE 6:**
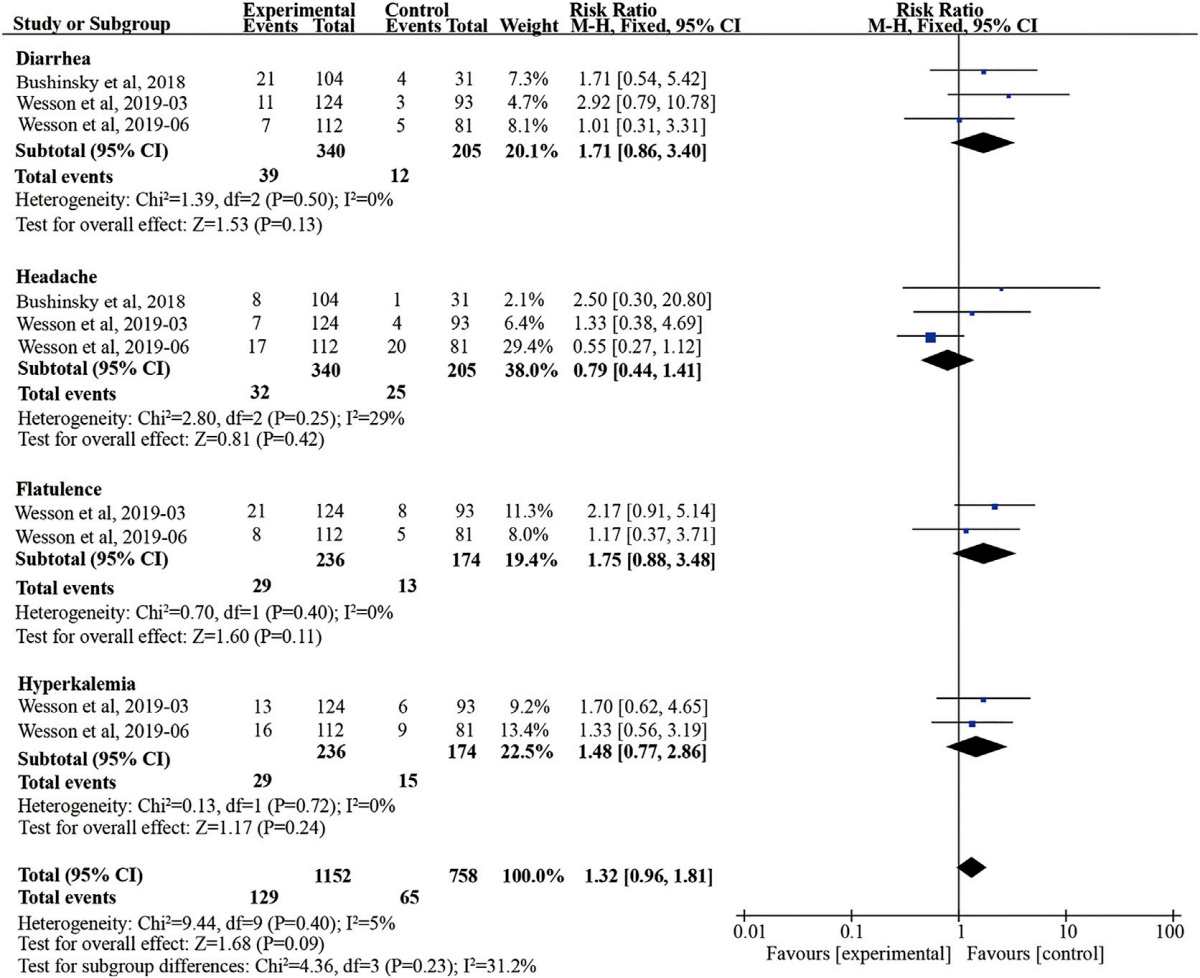
Forest plot for safety outcomes. M-H, Mantel-Haenszel method; 95% CI, 95% confidence interval.

Headache, diarrhea, flatulence, and hyperkalemia were considered the safety endpoints in our meta-analysis. In the three studies, veverimer was not associated with increased risk for developing treatment-emergent headache and diarrhea compared with the placebo group (diarrhea: RR 1.71, 95% CI [0.86, 3.40], *p* = 0.13, I^2^ = 0; headache: 0.79, 95% CI [0.44, 1.41], *p* = 0.42, I^2^ = 29%) (Figure 6). Also, there are two studies (Wesson et al., 2019a; Wesson et al., 2019b) reporting the risk of flatulence and hyperkalemia, but no difference between the two groups was observed (flatulence: RR 1.75, 95% CI [0.88, 3.48], *p* = 0.11, I^2^ = 0; hyperkalemia: RR 1.48, 95% CI [0.77, 2.86], *p* = 0.24, I^2^ = 0). Besides, GFR decreased and influenza also were discovered in some cases, but they did not have an effect on safety outcome (Bushinsky et al., 2018; Wesson et al., 2019a). Notably, both groups had similar endpoints of adverse events (*p* > 0.05) (Figure 6).

## Discussion

Our study pooled 548 individuals with acidosis associated with CKD from three high quality RCTs. The analysis demonstrated that veverimer use was effective against acidosis in patients with CKD, with a mean increase in serum bicarbonate level of by an average of 3.08 mmol/L compared with placebo, and was related to improved physical function. Besides, the pooled results indicated that veverimer use was not associated with an increased risk of developing adverse events. Heterogeneity analysis of all the included outcomes was low, indicating a high level of clinical evidence. The observed outcomes strongly support the use of veverimer is beneficial for the treatment of acidosis related to CKD.

Patients with acidosis usually have no obvious clinical symptoms and are aware of their acid-base disorder through a chemistry panel. In the chronic renal insufficiency cohort study, it was shown that patients with less than 22 mmol/L had an almost 2-fold increased risk of CKD progression (Dobre et al., 2015). Notably, it is well known that eGFR is a critical diagnostic criterion for the diagnosis of CKD (Goldenstein et al., 2014). Extensive research has provided clinical evidence that patients with CKD with impaired GFR are likely to develop metabolic acidosis. Moreover, studies have revealed that an increase in bicarbonate concentration to the normal range can delay renal replacement therapy in patients with CKD and slow down the decline in the eGFR (Di Iorio et al., 2019).

Since metabolic acidosis can lead to low function of multiple organs in the body, we need to focus on the treatment of acidosis. The National Kidney Foundation guidelines recommend maintaining the value of serum bicarbonate concentration to ≥22 mmol/L (Bailie and Massry, 2005). First-line therapy of metabolic acidosis includes reducing acidic foods intakes or using alkalization treatments that maintain total serum carbon dioxide (Susantitaphong et al., 2012). In available data from 20 patients with metabolic acidosis, Matthew K. et al. found that for every 0.1 mmol/kg increase in the daily dose of oral sodium bicarbonate treatment during two week periods, serum bicarbonate increased by 0.33 mmol/L (Abramowitz et al., 2013). Besides, Lambers et al. showed the dietary intervention also reduced the renal acid load of the patients to varying degrees (Lambers Heerspink et al., 2012). However, studies revealed that sodium bicarbonate therapy had been found to introduce additional sodium into the body, which may increase the potential risk of sodium-sensitive comorbidities such as edema and hypertension, making it necessary for patients to take diuretics and antihypertensives to mitigate the inevitable complications. Furthermore, previous studies revealed an undesirable effect of alkalinization therapy on cardiovascular events (Kendrick et al., 2018; Raphael, 2019; Klaerner et al., 2020). Moreover, alkalization treatments may reduce the effect of nephroprotective agents, indicating that traditional treatments may aggravate the deterioration of renal function (Lambers Heerspink et al., 2012; Chen and Abramowitz, 2019; Navaneethan et al., 2019).

Veverimer, as a hydrochloric acid adhesive, can effectively combine with and remove hydrochloric acid from the gastrointestinal tract, thereby increasing the concentration of bicarbonate without causing hypervolemia and fluid loss. Therefore, chloride removal by veverimer may be more suitable for CKD patients with various organic diseases. KDQoL-PFD is a widely used questionnaire based on the quality of daily life and the physical function of patients with kidney disease. It was demonstrated that both alkalization therapy and veverimer could improve KDQoL-PFD (Abramowitz et al., 2013; Witham et al., 2015). According to the report, the clinical difference of KDQoL subscales is at least 3–5 points before and after treatment (Samsa et al., 1999; Clement et al., 2009; Collister et al., 2016). In our pooled analysis, the average change in the veverimer group was 6.4 points, and that in the placebo group was only 1.1 points, with a significant intergroup difference of 5.25 (95% CI [1.58, 8.92], *p* < 0.01).

Nevertheless, the treatment group improved by 1.8 s on average in the repeated chair stand test, which was also used to assess physical function. This test score was similar to the minimum clinical difference value, indicating that veverimer cannot significantly extend the time of repeating chair stand test (WMD 0.84, 95% CI [−0.73, 2.40], *p* = 0.29). Based on the current clinical score, we speculate that this finding may be related to the sample size and the fact that the repeated chair stand test was in only two trials, thereby limiting the judgment regarding the effect of veverimer treatment on physical function. Notably, because of the lack of trials comparing alkalization and veverimer interventions, further clinical trials are needed to evaluate the clinical advantage of veverimer over traditional treatments.

We also further analyzed safety outcomes. Most often, primary attention is given to the diagnosis and treatment of diseases in clinical practice, but the possibility of adverse events is neglected, resulting in complications and severe harm to patients. Diarrhea, headache, flatulence, and hyperkalemia are common clinical adverse reactions and therefore were considered the safety endpoints of our assessment as well. Some studies have revealed that veverimer may cause severe gastrointestinal reactions, especially diarrhea (Klaerner et al., 2020). In our study, the veverimer treatment group did not exhibit any significant adverse reactions compared with the control group.

This is the first meta-analysis study regarding veverimer treatment in patients with CKD and metabolic acidosis to the best of our knowledge. The heterogeneity yielded low for all the included outcomes, indicating a high level of clinical evidence. Nonetheless, it still had shortcomings. First, there was a difference in traits (such as appearance) between the veverimer and placebo groups, and the site staff members could not fully implement the double-blind method. Second, the baseline bicarbonate level and eGFR in the veverimer group were slightly higher than those in the placebo group (Chen and Abramowitz, 2019). However, as a risk element for metabolic acidosis, the eGFR may make it easier for patients in the veverimer group with a slightly higher baseline to achieve the outcome of the evaluation, thereby increasing the heterogeneity of the study. Finally, the trial period of this study was relatively short, and the completed long-term clinical trial was only extended to 52 weeks. Therefore, further studies that could improve on the current data are warranted to evaluate the efficacy and safety of veverimer.

## Conclusion

The available clinical evidence indicates that veverimer treatment can be effective and safe in patients with CKD and may correct metabolic acidosis. Nonetheless, multicenter RCTs and large-scale trials are still needed to support this clinical evidence.

## Data Availability

The original contributions presented in the study are included in the article/Supplementary Material, further inquiries can be directed to the corresponding author.
